# Impact of Radiation Dose on Postoperative Complications in Esophageal and Gastroesophageal Junction Cancers

**DOI:** 10.3389/fonc.2021.614640

**Published:** 2021-03-10

**Authors:** Noah Kastelowitz, Megan D. Marsh, Martin McCarter, Robert A. Meguid, Narine Wandrey Bhardwaj, John D. Mitchell, Michael J. Weyant, Christopher Scott, Tracey Schefter, Priscilla Stumpf, Stephen Leong, Wells Messersmith, Christopher Lieu, Alexis D. Leal, S. Lindsey Davis, William T. Purcell, Madeleine Kane, Sachin Wani, Raj Shah, Hazem Hammad, Steven Edmundowicz, Karyn A. Goodman

**Affiliations:** ^1^Stanford University School of Medicine, Stanford, CA, United States; ^2^University of Colorado School of Medicine, Aurora, CO, United States; ^3^Icahn School of Medicine at Mount Sinai, New York, NY, United States

**Keywords:** esophageal cancer, gastro-esophageal junction cancer, chemoradiation, radiotherapy, esophagectomy

## Abstract

**Introduction:** The impact of radiation prescription dose on postoperative complications during standard of care trimodality therapy for operable stage II-III esophageal and gastroesophageal junction cancers has not been established.

**Methods:** We retrospectively reviewed 82 patients with esophageal or gastroesophageal junction cancers treated between 2004 and 2016 with neoadjuvant chemoradiation followed by resection at a single institution. Post-operative complications within 30 days were reviewed and scored using the Comprehensive Complication Index (CCI). Results were compared between patients treated with <50 Gy and ≥ 50 Gy, as well as to published CROSS study neoadjuvant chemoradiation group data (41.4 Gy).

**Results:** Twenty-nine patients were treated with <50 Gy (range 39.6–46.8 Gy) and 53 patients were treated with ≥ 50 Gy (range 50.0–52.5 Gy) delivered using IMRT/VMAT (41%), 3D-CRT (46%), or tomotherapy IMRT (12%). Complication rates and CCI scores between our <50 Gy and ≥ 50 Gy groups were not significantly different. Assuming a normal distribution of the CROSS data, there was no significant difference in CCI scores between the CROSS study neoadjuvant chemoradiation, <50 Gy, or ≥ 50 Gy groups. Rates of pulmonary complications were greater in the CROSS group (50%) than our <50 Gy (38%) or ≥ 50 Gy (30%) groups.

**Conclusions:** In selected esophageal and gastroesophageal junction cancer patients, radiation doses ≥ 50 Gy do not appear to increase 30 day post-operative complication rates. These findings suggest that the use of definitive doses of radiotherapy (50–50.4 Gy) in the neoadjuvant setting may not increase post-operative complications.

## Introduction

Neoadjuvant chemoradiation followed by esophagectomy is the standard of care for operable stage II-III esophageal and gastroesophageal junction cancers (1–3). Although there was concern that this trimodality therapy would increase the risk for post-operative complications, when compared to surgery alone, the addition of neoadjuvant carboplatin and paclitaxel with concurrent radiation therapy to 41.4 Gy was not shown to increase post-operative complication severity in the CROSS study group data (4). Radiation therapy doses of >41.4 Gy have historically been used in the United States for both definitive chemoradiation and in the neoadjuvant chemoradiation setting and could potentially improve outcomes, particularly for patients who are ultimately not able to undergo surgical resection. However, there has been concern that the higher doses may also be associated with higher toxicities, poorer surgical candidacy, or greater post-operative complication rates (5, 6). Here, we evaluate 30 day post-operative complications in esophageal and gastroesophageal junction cancer patients treated with trimodality therapy using radiation doses from 39.6 to 52.5 Gy at a single institution. We apply the same Comprehensive Complication Index (CCI) used to evaluate the post-operative complication severity in the CROSS study group data, and compare between patients treated with <50 Gy and ≥ 50 Gy, as well as to the prior CROSS study group results.

## Materials and Methods

### Study Population

Patients with esophageal or gastroesophageal junction cancers treated with neoadjuvant chemoradiation followed by surgical resection between 2004 and 2016 were identified through a retrospective systematic review of the electronic medical record at the University of Colorado Cancer Center. Patients were excluded if they were <18 years old, >89 years old, did not undergo subsequent surgical resection, or had incomplete follow up records for 30 days post resection.

### Patient Variables and Analysis

Selected patient characteristics and treatment details were recorded including age, sex, tumor type (adenocarcinoma vs. squamous cell carcinoma), chemotherapy type (carboplatin/paclitaxel vs. cisplatin/5-FU vs. other), radiation therapy method (intensity modulated radiotherapy [IMRT]/volumetric-modulated arc therapy [VMAT] vs. three-dimensional conformal radiation therapy [3D-CRT] vs. tomotherapy IMRT), and surgical resection type (Ivor Lewis esophagectomy vs. esophagogastrectomy vs. trans-hiatal approach).

Patients were grouped by radiation treatment dose, <50 Gy vs. ≥ 50 Gy, and clinical records were reviewed during the 30 day post resection period. Complications were categorized by type, graded based on the Clavien-Dindo classification, and CCI scores were calculated as previously described (4). In brief, for each complication a Clavien-Dindo classification was assigned based on deviation from a normal post-operative course and the required treatment/intervention (7). A CCI score was then calculated using all the Clavien-Dindo complication classifications for that single patient (8). CCI scores range from 0, indicating no complications, to 100 indicating patient death. The CCI calculation was accomplished using the online tool available at www.assesssurgery.com as previously described (8).

Comparison to previously published CROSS study neoadjuvant chemoradiation data (161 patients) was accomplished by assuming a normal distribution of the published data where therefore mean = median and standard deviation ≈ interquartile range/1.35.

### Statistical Analysis

Statistical analyses were performed using Prism 6 (GraphPad, La Jolla, CA). Comparisons between categorical variables were performed using the two-tailed Fischer's exact test. Comparisons between continuous variables were performed using the two tailed *t*-test for two groups or ANOVA with Tukey's test for three groups. For all analyses, a *p* < 0.05 was considered to be statistically significant.

## Results

### Patient and Treatment Characteristics

A total of 82 patients fit criteria for inclusion in our analysis. Twenty-nine patients (35%) were treated with <50 Gy (mean 44.8 Gy, range 39.6–46.8 Gy) and 53 patients (65%) were treated with ≥ 50 Gy (mean 50.4 Gy, range 50.0–52.5 Gy). Patient characteristics, age, sex, and tumor type, were not significantly different when comparing the <50 Gy and ≥ 50 Gy treatment groups (Table 1). Chemoradiation chemotherapy regimens and surgical resection methods were also similar (Table 1). A similar proportion of patients were treated with minimally invasive surgical approaches, 10 patients (34%) in the <50 Gy group vs. 23 patients (43%) in the ≥ 50 Gy, (*p* =0.49). A greater proportion of patients in the <50 Gy group received radiation treatment with 3D-CRT, that is, 66% compared to 36% in ≥ 50 Gy group (*p* =0.01). Conversely, more patients in the ≥ 50 Gy group received radiation treatment with IMRT/VMAT (53%) than in the <50 Gy group (36 %, *p* = 0.01).

**Table 1:** Patient and treatment characteristics.

	**Radiation dose**		
**Characteristic**	** <50 Gy**	**≥ 50 Gy**	***p-*value**
**Age—years**			
Mean, Range	63, 41–75	61, 34–78	0.48
Male:Female	27:2	44:9	0.31
**Tumor type—no. (%)**			
Adenocarcinoma	26 (90)	50 (94)	0.66
Squamous cell carcinoma	3 (10)	3 (6)	0.66
**Concurrent chemotherapy—no. (%)**			
Carboplatin/paclitaxel	14 (48)	35 (66)	0.16
Cisplatin/5-FU	6 (21)	8 (15)	0.55
Other	9 (31)	10 (19)	0.28
**Radiation method—no. (%)**			
IMRT/VMAT	6 (21)	28 (53)	**0.01**
3D-CRT	19 (66)	19 (36)	**0.01**
Tomotherapy IMRT	4 (14)	6 (11)	0.74
**Surgical resection—no. (%)**			
Ivor Lewis esophagectomy	20 (69)	41 (77)	0.44
Esophagogastrectomy	3 (10)	6 (11)	1.00
Trans-hiatal	6 (21)	6 (11)	0.33

*Bold highlights p value <0.05*.

### Complications and Pathologic Complete Response

There was no significant difference in the frequency of any of the assessed complication categories between the patients treated with <50 Gy or ≥ 50 Gy (Table 2). Pulmonary complications were the most common type of complication in both treatment groups. Rates of 30-day post-operative mortality were similar (10 vs. 6%, *p* = 0.66) and caused by a multifactorial combination of pulmonary (67%), gastrointestinal (50%), cardiac (33%), and infectious (33%) complications. The percentage of patients in each group who had any complication was also not statistically different (62 vs. 57%, *p* = 0.65). Complication grade rates were also very similar, with the only significant difference in Clavien-Dindo Grade IIIa complications (10 vs. 0%, *p* = 0.04). CCI scores did not differ between the two groups (27.21 ± 32.00 vs. 25.28 ± 29.74, *p* = 0.79). Rates of pathologic complete response were not significantly different (28 vs. 28%, *p* = 1.00).

**Table 2:** Post-operative events and radiation dose.

	**Radiation dose**		
**Postoperative events**	** <50 Gy**	**≥ 50 Gy**	***p-*value**
**Complications—no. (%)**			
Anastomic leakage	3 (10)	8 (15)	0.74
Pulmonary complications	11 (38)	16 (30)	0.62
Cardiac complications	5 (17)	10 (19)	1.00
Thromboembolic events	1 (3)	0 (0)	0.35
Wound infections	3 (10)	9 (17)	0.53
Death	3 (10)	3 (6)	0.66
**Clavien-dindo classification—no. (%)**			
Any	18 (62)	30 (57)	0.65
Grade I	4 (14)	7 (13)	1.00
Grade II	6 (21)	12 (23)	1.00
Grade IIIa	3 (10)	0 (0)	**0.04**
Grade IIIb	1 (3)	8 (15)	0.15
Grade IVa	3 (10)	3 (6)	0.66
Grade IVb	2 (7)	6 (11)	0.71
Grade V	3 (10)	3 (6)	0.66
CCI—mean ± SD	27.21 ± 32.00	25.28 ± 29.74	0.79
Pathologic complete response—no. (%)	8 (28)	15 (28)	1.00

### Comparison to CROSS

Comparison to published CROSS study group data for patients on the neoadjuvant chemoradiation arm showed a lower rate of pulmonary complications in our groups (*p* = 0.03) with otherwise similar rates of complications (Table 3). Mean CCI scores and pathologic complete response rates were not significantly different (Table 3).

**Table 3:** Comparison to post-operative events in CROSS study group data.

	**Radiation dose**			
**Postoperative events**	**<50 Gy**	**≥ 50 Gy**	**CROSS (41.4 Gy)**	***p-*value**
**Complications—no. (%)**				
Anastomic leakage	3 (10)	8 (15)	37 (23)	0.18
Pulmonary complications	11 (38)	16 (30)	81 (50)	**0.03**
Cardiac complications	5 (17)	10 (19)	34 (21)	0.86
Thromboembolic events	1 (3)	0 (0)	6 (3)	0.36
Wound infections	3 (10)	9 (17)	18 (11)	0.51
Death	3 (10)	3 (6)	8 (5)	0.52
CCI—mean ± SD	27.21 ± 32.00	25.28 ± 29.74	26.22 ± 18.63	0.93
Pathologic complete response—no. (%)	8 (28)	15 (28)	47 (29)	0.98

## Discussion

The treatment of esophageal cancer carries significant risk of morbidity and mortality. Trimodality therapy has been established as a standard of care for operable stage II-III esophageal and gastroesophageal junction cancers based on the results of the Phase III Randomized CROSS Trial comparing surgery alone vs. preoperative carboplatin and paclitaxel once weekly for 5 weeks with concurrent radiotherapy (41.4 Gy in 23 fractions), followed by transthoracic esophagectomy or transhiatal esophagectomy for gastroesophageal junction cancers (8). Using a lower radiotherapy dose than has been standard in the United States for neoadjuvant or definitive chemoradiation, the CROSS Study group demonstrated that the neoadjuvant chemoradiation is associated with similar post-operative complications as compared to surgery alone (4). The selection of the lower radiotherapy dose for the neoadjuvant chemoradiation in the CROSS Trial was based on prior studies showing a higher rate of post-operative mortality using higher doses of pre-operative radiotherapy and older treatment techniques (9, 10). However, there are no studies using modern radiotherapy techniques comparing the impact of using the more standard dose of 50–50.4 Gy to the lower dose of 41.4 Gy in the neoaduvant setting on post-operative complications. The CALGB 80803 trial evaluated induction chemotherapy followed by concurrent chemoradiation with a dose of 50.4 Gy and the toxicity has only been reported in abstract form, final results are pending (11).

In this retrospective study, we aimed to determine if higher radiation therapy doses during neoadjuvant chemoradiation increased 30 day post-operative complications. We found similar absolute rates of complications for patients treated with <50 Gy and ≥ 50 Gy, and CCI scores for the two groups were not different. Given that most patients who undergo intended trimodality therapy for esophageal and gastroesophageal junction cancers often have multiple co-morbidities making them non-ideal surgical candidates, many radiation oncologists are concerned that giving 41.4 Gy may not be an adequate dose in patients who may ultimately not undergo surgery after chemoradiation. These data are reassuring that the use of a more definitive dose of 50–50.4 Gy using modern radiation therapy techniques in the neoadjuvant setting does not appear to increase the risk of post-operative complications.

In a smaller study of 24 patients, Nabavizadeh et al. reported on their clinical experience using chemoradiation radiation doses > 41.4 Gy and noted a potential increased risk of severe radiation-induced acute lung injury with higher radiation doses (5). Here, we observed a similar rate of pulmonary complications in our <50 Gy and > 50 Gy groups, and lower rates overall than that of the CROSS study group. Direct interpretation of this difference is challenging as a greater proportion of the patients treated here with ≥ 50 Gy received radiation treatment with IMRT/VMAT rather than 3D-CRT, while all patients in the CROSS study group were treated with 3D-CRT. Compared to 3D-CRT, IMRT has been shown to reduce the percent volumes of the heart and lungs that are irradiated, which may explain the reduced pulmonary toxicity seen here as compared to the CROSS study group (12, 13). Higher radiation doses may also increase risk for late treatment toxicities, although clinical data suggests that IMRT can help reduce this risk, for example decreasing death from cardiac causes, as compared to treatment with 3D-CRT (14).

In theory, a higher radiation dose should also lead to greater pathologic complete response rates and possibly improved patient outcomes. Of note, we did not see an increase in rates of pathologic complete response with higher radiation dose. The optimal dose of radiotherapy in the setting of neoadjuvant chemoradiation for esophageal and gastroesophageal junction cancers is still debated and prospective data are needed to evaluate the impact on both the pathologic response rates as well as the post-operative complications. Limitations of this study are its retrospective nature and the relatively small sample size, making it difficult to definitively answer the question of the impact of radiation dose on either outcome or toxicity. Nonetheless, in the absence of prospective data, acceptable doses of radiotherapy in the neoadjuvant setting range from 41.4 to 50.4 Gy and the selection of dose may be based on multiple factors. For patients who are deemed good surgical candidates and have a high chance of going to surgery, 41.4 Gy is an appropriate dose. For patients where surgery is tentative based on overall performance status and co-morbidities, this data is reassuring that a definitive dose of radiotherapy, delivered with modern radiation therapy techniques, does not appear to increase the risk of post-operative complications should the patient ultimately be able to undergo surgery.

## Data Availability Statement

The raw data supporting the conclusions of this article will be made available by the authors, without undue reservation.

## Ethics Statement

The studies involving human participants were reviewed and approved by Colorado Multiple Institutional Review Board. Written informed consent for participation was not required for this study in accordance with the national legislation and the institutional requirements.

## Author Contributions

NK and KG study concepts, design, and data analysis. NK, MMa, MMc, RM, NB, JM, MW, CS, TS, PS, SL, WM, CL, AL, SD, WP, MK, SW, RS, HH, SE, and KG writing and/or editing the manuscript. All authors reviewed the manuscript before submission.

## Conflict of Interest

The authors declare that the research was conducted in the absence of any commercial or financial relationships that could be construed as a potential conflict of interest.
